# Wireless High-Frequency Peripheral Nerve Stimulation for Chronic Refractory Knee Pain Post-total Knee Replacement

**DOI:** 10.7759/cureus.35759

**Published:** 2023-03-04

**Authors:** Gaurav Chauhan, Suresh K Srinivasan, Suchit Khanduja

**Affiliations:** 1 Anesthesiology and Perioperative Medicine, University of Pittsburgh Medical Center Presbyterian, Pittsburgh, USA; 2 Pain Management, Trinity Medical Center West, Steubenville, USA; 3 Anesthesiology, Beaumont Hospital, Royal Oak, USA

**Keywords:** chronic post-surgical pain, chronic pain, wireless implantable device, peripheral nerve stimulation, total knee replacement

## Abstract

The number of joint replacement surgeries, especially knee replacement surgeries, is rising with the rising geriatric population. Chronic unremitting knee pain post-total knee replacement surgery is a common phenomenon. Usually, the pain responds to conservative measures, including physical therapy and medical management. In some patients, the pain post-knee replacement surgery can be refractory and unremitting. In such scenarios, peripheral nerve stimulation, or neuromodulation, can be an effective option.

## Introduction

Total knee replacement (TKR) surgery is highly effective for relieving pain and improving function in individuals with knee osteoarthritis. According to recent data, the number of TKR surgeries in the United States has increased dramatically. In 2020, over 700,000 TKR surgeries were performed in the United States [1]. This represents a significant increase from previous years and is primarily due to an aging population and advances in surgical techniques and implant design [1].

Most patients undergoing TKR surgery experience significant pain relief and improved function. According to some studies, up to 20% of people may experience pain after a TKR [2,3]. The presence of pain after TKR does not necessarily mean the surgery was not successful. Further treatment, such as physical therapy or revision surgery, may be necessary to alleviate ongoing pain. Furthermore, other factors, such as implant failure, infection, or nerve damage during surgery. In some cases, persistent pain may be related to the underlying condition that led to the need for knee replacement, such as osteoarthritis [4-6].

Multiple treatment modalities are available for the treatment of knee pain post-TKR. Physical therapy is one of the most effective methods, as it helps improve strength, flexibility, and range of motion in the knee, thereby reducing pain and improving function [7,8]. Over-the-counter pain relievers, such as ibuprofen or acetaminophen, can help manage knee pain. Assistive devices, such as a cane or knee brace, can reduce stress on the knee and relieve pain. Applying ice or heat to the knee can help reduce swelling and relieve pain. Maintaining a healthy weight and avoiding high-impact activities that put excessive stress on the knee can also help manage pain [9].

However, in some cases, knee pain post-TKR could be refractory to multiple treatment modalities, including surgery. In recent years peripheral nerve stimulation (PNS) devices have yielded significant efficacy for pain and associated symptoms in patients with chronic refractory knee pain post-TKR. The authors present one such challenging case that reports the successful use of wireless high-frequency PNS for chronic refractory knee pain post-TKR.

## Case presentation

A 72-year-old male patient that consented to the case report presented to our clinic with a 12-year history of bilateral chronic knee pain. The patient had a body mass index of 32 kg/m² and had a history of controlled hypertension. The patient underwent TKR surgery four years back, followed by two revision surgeries on the right knee within two years of the initial surgery. The patient reported diffuse camping and sharp ache in the right knee that worsened over the years. The patient was scheduled to undergo physical therapy for six months after each surgery but couldn’t complete the whole duration due to incessant pain. The patient had failed gabapentin (1200 mg TID), Cymbalta, various non-steroidal anti-inflammatory agents, topical diclofenac gel, lidocaine 4% and 5% patches, transcutaneous electrical stimulation therapy, and genicular nerve radiofrequency ablation.

The patient was referred to the authors’ clinic for spinal neuromodulation. The patient was currently on Lyrica 200 mg three times daily, nabumetone 1000 mg twice daily, memantine 10 mg twice daily, oxycodone 10 mg three times daily as needed, and nortriptyline 75 mg at bedtime. The patient reported 5-6/10 pain on the numeric rating scale (NRS) at rest and 6-8/10 on the NRS scale upon walking or getting up from a sitting position. The physical exam reported severe pain on flexion-extension of the right knee, which precluded further exam. The patient was ambulating with a walker for the last three years and reported that due to knee pain, he has a predominantly sedentary lifestyle. Subsequent imaging of the right knee and a review of the computerized tomographic scan of the right knee reported intact hardware.

**Figure 1:**
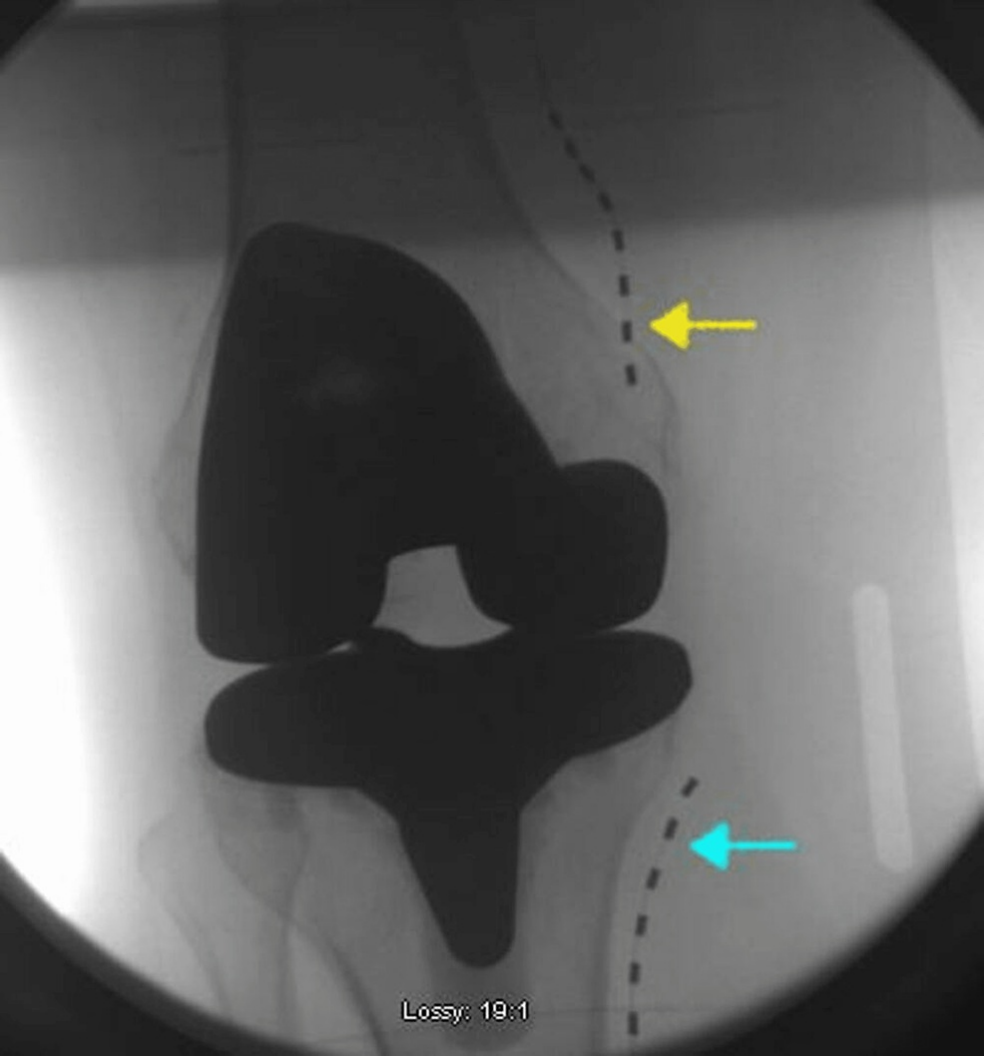
Anteroposterior fluoroscopic image of the right knee with Stimwave peripheral nerve stimulator leads Yellow arrow: superomedial trial lead for stimulation of superomedial genicular nerves Blue arrow: inferomedial trial lead for stimulation of inferomedial genicular nerves

The patient was counseled about the Stimwave Peripheral Nerve Stimulator therapy for refractory knee pain. The patient underwent a Stimwave trial, with lead placement, under fluoroscopic guidance, at the anatomic location of superomedial and inferomedial genicular nerves (Figure 1).

The leads were stimulated in tandem, and the patient reported significant relief from the superomedial leader, placed along the medial femoral condyle. During the provisional seven-day test period the patient reported more than 80% improvement in pain and associated symptoms with activation of superomedial femoral lead. At the end of the trial week, the PNS leads were removed.

**Figure 2:**
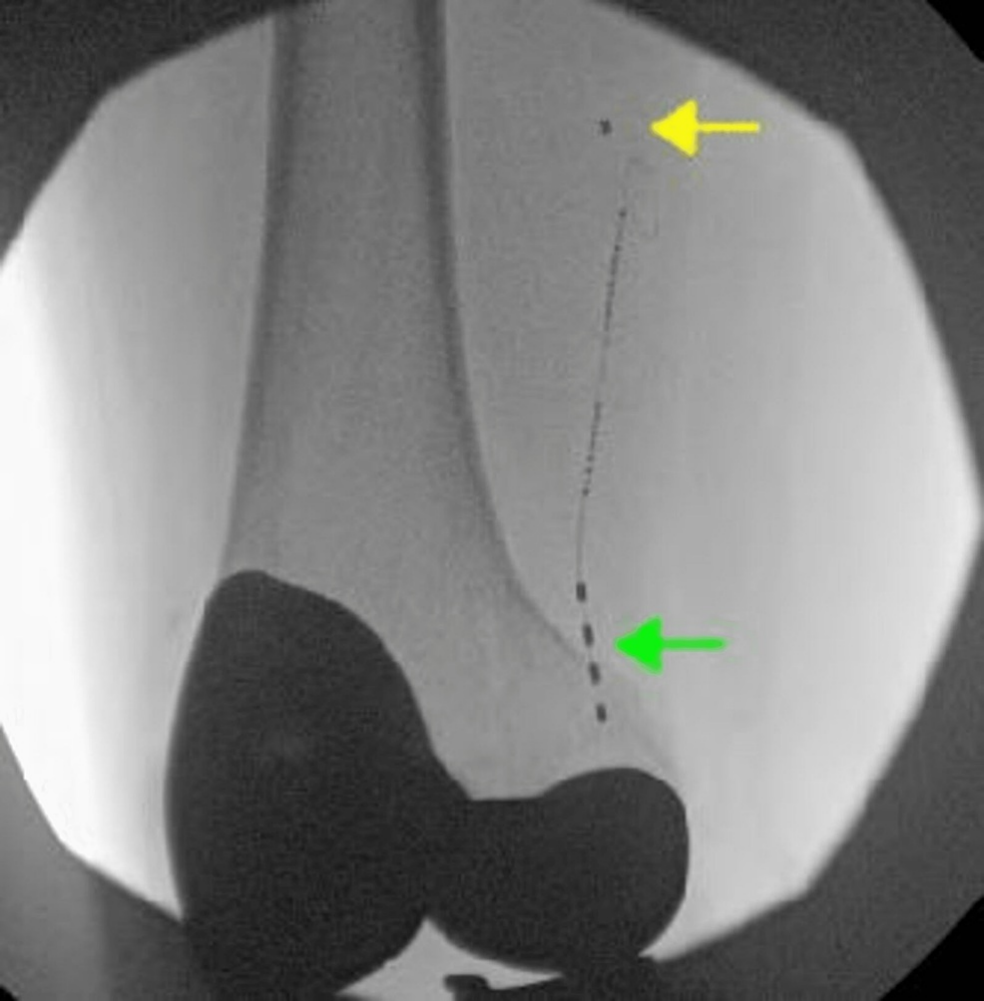
Anteroposterior fluoroscopic image of permanently implanted superomedial stimwave lead Green arrow: quadripolar lead for stimulation of superomedial genicular nerves Yellow arrow: lead is looped and secured in a superficial tissue pocket

The patient was scheduled for permanent lead placement along the anatomical location of superomedial genicular nerves. A 12-gauge introducer needle was placed under fluoroscopic guidance along the medial condyle of the right femur. The four lead PNS lead was placed through the electrode and was advanced along the medial condyle of the femur. Subsequently, the introducers were removed along with the blank stylet of the PNS lead. The radio receiver stylet was placed in the lead. Stimulation was then tested to capture the patient’s region of pain in the right knee. At this point, a small pocket was made proximally to the initial incision sites. At this point, the lead was then tunneled from their initial entry site incisions to the proximal tissue pockets using 13-gauge introducers (Figure 2).

The patient reported minimal pain after the procedure. At six months post-procedure, the patient continues to report >80% improvement in his knee pain and associated symptoms. The patient has weaned off opioids but continues the rest of his medications at the same dosage before the PNS implant. The patient reports a 0/10 pain score at rest and a 2-3/10 pain score upon getting up from a sitting position and during ambulation. The patient is tolerating physical therapy and is able to achieve his goal of walking for longer distances.

## Discussion

The use of PNS for knee pain involves the placement of electrodes near the peripheral nerves that transmit pain signals to the brain. Intimate placement of stimulation leads and subsequent stimulation parameters are designed to activate Aα/β fibers while avoiding smaller pain fibers (Aδ/C fiber). This form of selective remote stimulation is hypothesized to close the gate for afferent pain-carrying signals to the brain and is the most widely accepted theory of the mechanistic model of PNS [10-13]. Various reports have commented on the efficacy of PNS as an alternative to traditional forms of pain management, such as opioid medications, for treating chronic knee pain [14]. The benefits of PNS for knee pain include minimal invasiveness, low risk of side effects, and long-term pain relief for many patients [14,15].

Stimwave's wireless PNS technology (StimwaveTechnologies, Pompano Beach, FL, USA) has been approved by the U.S. Food and Drug Administration (FDA) for the treatment of chronic intractable pain in the lower back, neck, knee, and other parts of the body. As far as peripheral stimulation devices go, Stimwave is unique as it lacks a bulky external battery. The pulse generator is located behind the electrode and is part of the lead. Any electrode can be positive or negative, allowing for the electric arc/circuit to complete at the level of the electrodes. This allows the higher frequency to be tolerated without any sensation at the skin level. The battery is worn externally over the distal end of the lead. It communicates to the lead via a receiver radiofrequency wire inserted inside the electrode at the time of implant. The nature of the chronic pain condition and the intended target can help plan the insertion site and tunnel the tail end. At least 23 cm of the lead must be implanted, and the distal end (beyond the receiver marker band) can be cut. The internal lead is compatible with the entire body magnetic resonance imaging at 1.5 Tesla [16,17].

There are multiple case reports of Stimwave PNS for other chronic pain syndromes [18-20]. However, there is not enough data to advocate the use or to comment on the placement of this device in treatment algorithms in cases of refractory, chronic pain post-TKR. As the design of the PNS system undergoes a rapid evolution and becomes more ergonomic, physicians can seek to offer these therapies earlier in the treatment algorithm for chronic refractory pain post-TKR.

## Conclusions

Stimwave's wireless PNS technology can be considered in patients with chronic unremitting pain post-TKR. The data is limited regarding the place of PNS in the treatment algorithm for such cases. The long-term efficacy of PNS is still under review, and the field needs more high-quality research trials to outline the short- and long-term adverse effects. Rapid advancement in the field of PNS, with respect to ease of insertion and enhanced comfort while using these devices, begets the question if physicians should offer this therapy, if indicated, earlier in the treatment algorithm for refractory chronic pain conditions. The authors envision an increased use of PNS devices in the future, guided by robust evidence-based literature.

## Figures and Tables

**Figure 3:**
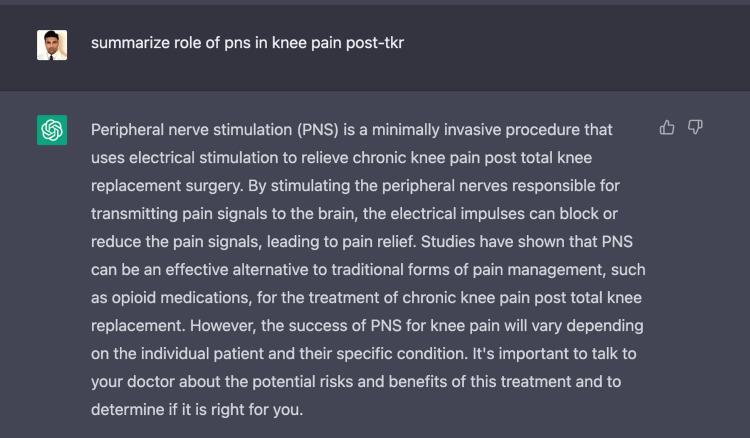
ChatGPT snapshot of the author
